# Combined IL6 and CCR2 blockade potentiates antitumor activity of NK cells in HPV-negative head and neck cancer

**DOI:** 10.1186/s13046-024-03002-1

**Published:** 2024-03-12

**Authors:** Fan Yang, Chenyang Yuan, Fanghui Chen, Zhaohui S. Qin, Nicole C. Schmitt, Gregory B. Lesinski, Nabil F. Saba, Yong Teng

**Affiliations:** 1 Department of Hematology and Medical Oncology, Winship Cancer Institute, Emory University, 201 Dowman Dr, Atlanta, GA 30322 USA; 2 Department of Biostatistics and Bioinformatics, Rolling School of Public Health, Emory University, Atlanta, GA 30322 USA; 3 Department of Otolaryngology, Emory University, Atlanta, GA 30322 USA; 4 Department of Oral Biology and Diagnostic Sciences, Georgia Cancer Center, Augusta University, Augusta, GA 30912 USA; 5 Wallace H. Coulter Department of Biomedical Engineering, Georgia Institute of Technology and Emory University, Atlanta, GA 30322 USA

**Keywords:** Head and neck cancer, NK cells, IL6 and CCR2, scRNA-seq, HPV, The tumor microenvironment, Double blockade

## Abstract

**Background:**

While T cell-activating immunotherapies against recurrent head and neck squamous cell carcinoma (HNSCC) have shown impressive results in clinical trials, they are often ineffective in the majority of patients. NK cells are potential targets for immunotherapeutic intervention; however, the setback in monalizumab-based therapy in HNSCC highlights the need for an alternative treatment to enhance their antitumor activity.

**Methods:**

Single-cell RNA sequencing (scRNA-seq) and TCGA HNSCC datasets were used to identify key molecular alterations in NK cells. Representative HPV-positive ( +) and HPV-negative (** −**) HNSCC cell lines and orthotopic mouse models were used to validate the bioinformatic findings. Changes in immune cells were examined by flow cytometry and immunofluorescence.

**Results:**

Through integration of scRNA-seq data with TCGA data, we found that the impact of IL6/IL6R and CCL2/CCR2 signaling pathways on evasion of immune attack by NK cells is more pronounced in the HPV** − **HNSCC cohort compared to the HPV + HNSCC cohort. In orthotopic mouse models, blocking IL6 with a neutralizing antibody suppressed HPV** − **but not HPV + tumors, which was accompanied by increased tumor infiltration and proliferation of CD161^+^ NK cells. Notably, combining the CCR2 chemokine receptor antagonist RS504393 with IL6 blockade resulted in a more pronounced antitumor effect that was associated with more activated intratumoral NK cells in HPV − HNSCC compared to either agent alone.

**Conclusions:**

These findings demonstrate that dual blockade of IL6 and CCR2 pathways effectively enhances the antitumor activity of NK cells in HPV-negative HNSCC, providing a novel strategy for treating this type of cancer.

**Supplementary Information:**

The online version contains supplementary material available at 10.1186/s13046-024-03002-1.

## Background

Head and neck squamous cell carcinoma (HNSCC) is a form of cancer that originates from the squamous cells lining the mucous membranes within the head and neck region. It encompasses a spectrum of cancers that develop in structures including the oral cavity, pharynx, hypopharynx and larynx. Human papillomavirus (HPV) has been identified as a risk factor for specific types of HNSCC, particularly those affecting the oropharynx. Patients with HPV-positive ( +) HNSCC harbor distinct prognostic characteristics and genomic patterns compared to those with HPV-negative ( −) disease. Generally, HPV + HNSCC confers a more favorable prognosis compared to HPV − HNSCC [1]. Moreover, the immune system’s reaction to HPV + HNSCC can be more potent, leading to the improved efficacy of immunotherapy agents including immune checkpoint inhibitors (ICIs, *e.g.,* pembrolizumab and nivolumab) in the clinical setting [2–4]. Nevertheless, even with these advances, individuals with HPV + HNSCC still experience recurrent or metastatic (R/M) disease. HPV − HNSCC is often linked to a history of tobacco and alcohol use and tends to carry a less favorable prognosis. Targeted therapeutics for HPV − HNSCC, including agents such as cetuximab, are used to specifically target EGFR involved in cancer growth and metastasis. In addition, primary clinical data have revealed that the response rate to anti-PD-1 ICIs may be more dampened in HPV − patients [3, 5, 6].

While ICIs that activate T cells against recurrent HNSCC improve clinical outcomes for selected patients, they are often ineffective for the majority. Consequently, there is a growing inclination to explore alternative immune effector cells as potential targets for immunotherapeutic interventions. Natural killer (NK) NK cells contribute to the immune response against tumors by recognizing and attacking cancer cells, exhibiting characteristics aligned with both innate and adaptive immune responses [7]. Generally, human NK cells receive education through inhibitory signaling mediated by receptors like NKG2A or KIR, which bind to HLA-E or HLA-A, HLA-B, HLA-C, HLA-F, and HLA-G, respectively [8]. In the context of HNSCC, NK cells are important for tumor surveillance and control, and the tumor infiltration of NK cells has consistently been correlated with more favorable prognoses [9]. Deficiencies in HLA expression are frequently observed in HNSCC. HLA class I expression is lower in HPV + HNSCC compared to the HPV − counterpart [10, 11]. Interestingly, reduced HLA class I expression correlates with an improved prognosis in HPV + HNSCC, but a less favorable prognosis in HPV − HNSCC. This difference may be due to the relatively lower infiltration of NK cells in HPV − HNSCC, which may protect the tumors from the consequences of HLA loss.

**Fig. 1:**
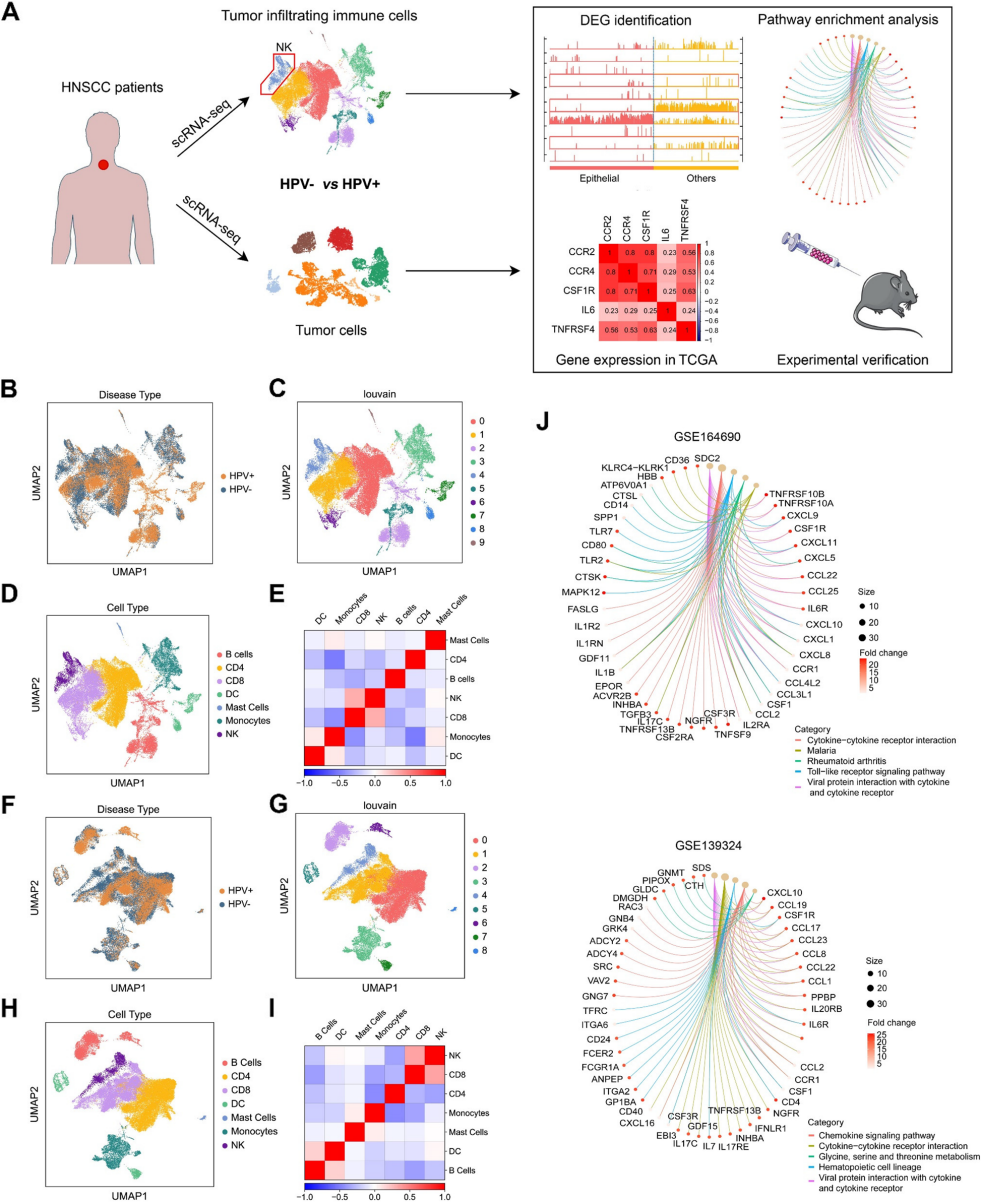
NK cell enrichment in HPV − HNSCC by scRNA-seq analysis. **A** Schematic showing analysis from bioinformatics to experimental validation. (B-E) Bioinformatic analysis of immune cells based on the scRNA-seq data from GSE164690. **B** Visualization of all analyzed cells stratified by HPV infection status using UMAP, colored by sample ID. **C** Visualization of all analyzed cells using UMAP, colored by HPV status. **D** Louvain clustering on all analyzed cells identified 10 cell clusters. **E** Correlation heatmap of all identified cell types. **F**-**I** Bioinformatic analysis of immune cells based on the scRNA-seq data from GSE139324. **F** Visualization of all analyzed cells stratified by HPV infection status using UMAP, colored by sample ID. **G** Visualization of all analyzed cells using UMAP, colored by HPV status. **H** Louvain clustering on all analyzed cells identified 9 cell clusters. **I** Correlation heatmap of all identified cell types. **J** Enriched pathways derived from tumor infiltrating NK cells (HPV − tumors *vs.* HPV + tumors) using the scRNA-seq data from GSE164690 (upper panel) and GSE139324 (lower panel)

By integrating single-cell RNA sequencing (scRNA-seq) data with TCGA HNSCC dataset, we identified differentially expressed genes (DEGs) and several enriched pathways within NK cells and tumor cells, depending on HPV infection status (Fig. 1A). Our computational analysis suggests that HPV − tumor cells have a stronger reliance on IL6/IL6R and CCL2/CCR2 signaling within the tumor microenvironment (TME) to evade NK cell immune attack, in contrast to HPV + tumors. In orthotopic tumor mouse models, we observed suppression of HPV − tumors upon inhibition of IL6, accompanied by increased tumor infiltration and proliferation of CD161^+^ NK cells. Notably, combining the CCR2 chemokine receptor antagonist RS504393 with IL6 blockade resulted in a more pronounced antitumor effect that was associated with more activated intratumoral NK cells in HPV − HNSCC compared to either agent alone. This combined approach represents a more effective strategy for the treatment of HPV − HNSCC.

## Materials and methods

### Data information

We analyzed two public datasets available in the Gene Expression Omnibus (GEO) database, GSE164690 [12] and GSE139324 [13]. For both datasets, we retained tumor-infiltrating immune cells from HNSCC patient samples. We also retained CD45- cells, which were only present in GSE164690. After filtering, GSE164690 contained 18 samples (12 HPV − samples and 6 HPV + samples) with 33,545 genes, 64,711 immune cells, and 48,868 non-immune cells. GSE139324 contained 26 samples (18 HPV − samples and 8 HPV + samples) with 33,694 genes and 60,932 immune cells. Detailed information on the patient cohorts is provided in Supplementary Table S1 and Table S2.

### Data filtering and pre-processing

To ensure the quality of the data for downstream analyses, we filtered out cells with less than 200 expressed genes, cells with more than 2,500 expressed genes, and cells with more than 5% mitochondrial genes. We also removed genes expressed in fewer than 5 cells [14]. Data processing steps followed the pipeline in the Python package SCANPY [15]. We scaled the expression values to 10,000 counts per cell and log-transformed the data. We then identified and retained highly variable genes (mean expression between 0.01 and 5, dispersion greater than 0.2) for further analysis. We then regressed out the effects of mitochondrial genes and total counts per cell using linear regression. Finally, we scaled the data to unit variance and clipped values exceeding standard deviation 10.

### Clustering and cell type identification

We first reduced the dimensionality of the data by Principal Component Analysis (PCA). Following the pipeline in SCANPY, we constructed a neighborhood graph based on the first 50 PCs of the data and performed Louvain clustering on the neighborhood graph [16]. The resolution was selected based on the Silhouette score of each clustering. We visualized the cell clusters on UMAP plots [17]. We identified cell types by measuring the expression level of a list of known marker genes in each cluster.

### DEGs and pathway enrichment analysis

We detected DEGs using the t-test with overestimated variance as part of the “rank_genes_group” function in SCANPY. We used 1.5 log fold change (logFC) and 0.05 *p*-value as cutoffs and ranked all DEGs by their logFC. We removed all long intergenic non-coding RNA and miscellaneous RNA. We performed pathway enrichment analysis based on the DEG lists using Gene Ontology (GO) and KEGG enrichment analysis tools.

### TCGA data analysis

Gene expression and clinical data were downloaded from TCGA HNSCC database and filtered into two subgroups with HPV − (410 cases) and HPV + (69 cases) [18]. Gene raw counts were performed to construct the ‘gene expression matrix’ with the gene symbol and normalized by using log2 (read counts + 1). Differences in IL6 expression between HPV − and HPV + HNSCC were compared using t-test. The single-gene GSEA analysis of IL6 was performed (log FC > 1.5, adjusted *p*-value < 0.05) among HPV − HNSCC. Pathways enrichment and gene correlation were evaluated in HPV − HNSCC cohort using R.

### Cell lines and culture

Mouse oral squamous cell carcinoma MOC2 cells (HPV −) were obtained from Kerafast (Boston, MA). Mouse TC-1 (HPV +) tumor cells were obtained as a kind gift from Dr. T.C. Wu of Johns Hopkins University and maintained as previously described [19]. All cells were used for experiments before passage 10 and cultured in complete DMEM medium (ThermoFisher Scientific) containing 10% FBS (Biological Industries) at 37 °C in a humidified incubator supplied with 5% CO_2_. All cell lines were routinely screened for mycoplasma contamination by MycoAlert Mycoplasma Detection Kit (Lonza).

### Animals and treatment

Six-week-old male C57BL/6 mice were purchased from the Jackson Laboratory (Bar Harbor, ME). All animal experiments were approved by the Institutional Animal Care and Use Committee (IACUC) of Emory University and Augusta University. Buccal mucosal tumors were established in C57BL/6 mice by intramucosal inoculation of 1.0 × 10^6^ MOC2 or TC-1 cells [19, 20]. Ten days after cell inoculation, tumor-bearing mice were randomized into two groups to receive murine anti-IL6 neutralizing antibody (αIL6, clone MP5-20F3, BioXCell) and IgG2 isotype. Mice were monitored daily for distress and tumor volumes were recorded every five days. For animal combination treatments, tumor-bearing mice were randomized to receive the treatment of IgG2 isotype, αIL6, RS504393 (Catalog No: HY-15418, MedchemExpress) or the combination of αIL6 and RS504393. αIL6 (100 μg/mouse) and/or RS504393 (6 mg/kg) were administered by intraperitoneal injection once every three days for a total of five doses. To deplete NK cells in C57BL/6 mice, 50 μg anti-Asialo GM1 antibody (αASGM1, ThermoFisher) or its control rabbit polyclonal IgG was administered by intraperitoneal injection once every three days for a total of five doses [21, 22]. To determine the involvement of NK cells in the antitumor activity of the combination of αIL6 and RS504393, MOC2 tumor-bearing mice were treated with αASGM1 one day before the first dose of αIL6 plus RS504393 and the same procedure as described above was repeated. All mice were maintained in an unbiased fashion and no mouse was excluded from the analysis.

### Tumor tissue preparation

Mice were euthanized with CO_2_, and tumor tissues and lymph nodes were collected, minced into small pieces, and digested in 2 mg/ml collagenase type IV (Catalog No: LS004188, Worthington-Biochem) at 37 °C for 1 h, followed by storage in cold PBS. Single cell suspensions of tumor tissues were prepared in PBS containing 10% FBS and 2 mM EDTA, and tissues were disrupted on the surface of a 40 µm cell filter using the plunger of a 1 ml syringe. Peripheral blood was collected by enucleation, and erythrocytes were lysed with ACK Lysing Buffer (ThermoFisher Scientific) to obtain a peripheral blood mononuclear cell suspension. Serum IL6 in mice was measured using an IL6 mouse ELISA kit (ThermoFisher Scientific).

### Flow cytometry analysis

Single cell suspensions of tumor tissue were stained with fluorescent dye-conjugated antibodies. Antibodies: CD3 (Catalog No: 560527, BD Biosciences, San Jose, CA), CD8 (Catalog No: 100722, BD Biosciences), CD4 (Catalog No: 560246, BD Biosciences), CD161 (Catalog No: 108706, BD Biosciences), and Ki67 (Catalog No: 12–5698-82, ThermoFisher Scientific). All experiments were performed on FACSCalibur and FACSAria IIIu (BD Biosciences) and analyzed using FlowJo 10.0 software (BD Biosciences).

### Immunofluorescence staining

MOC2 and TC-1 tumor tissue was fixed with 4% formaldehyde in PBS for 15 min and permeabilized with 0.1% Triton X-100 in PBS for 10 min. After blocking for 1 h, tumor sections were incubated with anti-CD161 antibody at 4 °C overnight, followed by incubation with Alexa Flour 488-conjugated secondary antibody (Abcam, Cambridge, United Kingdom) in the dark for 1 h. Slides were mounted with Vectashield mounting medium (Vector Laboratories, Burlingame, CA) containing the DAPI nuclear stain before examination under a fluorescence microscope. Fluorescent images were captured using an inverted fluorescence microscope (BZ-X710 All-in-one, Keyence). The quantitation of positive CD161 cells in the tumor sections was counted in at least 10 random fields and based on the results provided by three investigators who were blind to treatment information.

### Statistical analysis

Tumor volume sizes were recorded according to tumor growth time and weighted after tumor tissues were harvested. Data were analyzed using Statistical software GraphPad Prism 9 (San Diego, CA). Experimental values are expressed as mean ± standard deviation (SD). For comparison between two groups, statistical analysis was performed using unpaired Student’s *t*-test. Tumor volume was modeled over time using mixed-effects regression with fixed effects for group, time, and the interaction between them. Random intercepts and slopes by mouse were included with an unstructured covariance matrix for random effects. Other outcomes were compared using one-way analysis of variance (ANOVA). *p* values less than 0.05 were considered statistically significant.

## Results

### Specific NK cell enrichment in HPV − HNSCC revealed by scRNA-seq analysis

We first analyzed a public scRNA-seq dataset (GSE164690) containing tumor-infiltrating immune cells from a cohort of 18 patients, including 12 patients with HPV- HNSCC and 6 patients with HPV + HNSCC. After basic filtering and pre-processing, the cells were visualized on a 2D plot using the Uniform Manifold Approximation and Projection (UMAP) technique. Basic clinical information is represented by colors of the cells based on the sample ID and HPV status of the patients. As depicted in the plots, the samples mixed well with negligible batch effect (Supplementary Fig. S1A). However, the distribution of HPV − and HPV + samples displayed a noticeable and non-random arrangement in certain specific regions (Fig. 1B), suggesting that HPV − samples could be concentrated within particular cell types, while HPV + samples might exhibit enrichment in other cell types.

Louvain clustering was performed on all analyzed cells and a total of 10 clusters were identified (Fig. 1C). To identify the corresponding cell type of each cluster, we examined the expression level of a set of marker genes in each cluster. Employing both visualization techniques and quantification methods, we attributed each cluster with a cell type label based on the highest expression of marker genes associated with that specific cluster. Through this process, we successfully categorized a total of 7 distinct cell types: CD4 T cells (cluster 0), CD8 T cells (clusters 1 and 6), B cells (clusters 2 and 5), monocytes (cluster 3), NK cells (cluster 4), dendritic cells (clusters 7 and 8), and mast cells (cluster 9) (Fig. 1D). We validated these procedures by generating a correlation heatmap across all cell types, where the presence of low correlations between different cell type pairs indicated the accuracy of clustering and successful identification of cell types (Fig. 1E).

To counterbalance the uneven distribution of HPV − and HPV + samples across all cells, we computed the proportion of HPV − and HPV + samples within each individual cluster. We found that, except for cluster 0 and cluster 1, all clusters exhibited varying imbalances in the numbers of HPV − and HPV + samples. Notably, among the clusters with elevated HPV − samples, cluster 4 showcased the most prominent deviation, with the proportion of HPV − samples markedly surpassing the overall cohort-level proportion (Supplementary Fig. S1B). The UMAP plot depicting all cells and color-coded according to HPV status echoed this observation, highlighting that cluster 4 predominantly consisted of HPV − samples. Aligning with our prior cell type identification, cluster 4 was assigned to NK cells. Upon further analysis of scRNA-seq data in GSE164690, it was observed that NK cells in cluster 4 exhibited a higher proportion in HPV − samples in comparison to HPV + samples (Supplementary Fig. S1B).

Subsequently, we analyzed another publicly available scRNA-seq dataset (GSE139324), also comprising tumor-infiltrating immune cells. This dataset encompassed a cohort of 26 patients, including 18 HPV − and 8 HPV + HNSCC patients. We executed the same filtering and pre-processing steps as in the previous analysis to ensure consistency. Similarly, UMAP representation indicated no prominent batch effect (Supplementary Fig. S1C), yet specific regions manifested an uneven distribution of HPV − and HPV + samples (Fig. 1F). Louvain clustering was subsequently applied to all processed cells using the same clustering resolution, yielding the identification of 9 distinct clusters (Fig. 1G). By employing the same set of marker genes and procedures described above, we successfully attributed cell type labels, yielding consistent cell type assignments: CD4 T cells (cluster 0), CD8 T cells (cluster 1), B cells (clusters 2 and 6), monocytes (cluster 3), NK cells (cluster 4), dendritic cells (clusters 5 and 7), and mast cells (cluster 8) (Fig. 1H and I). Notably, cluster 4 in this dataset, identified as NK cells, showed a similar unbalanced distribution in HPV- and HPV + samples, with a notable overrepresentation of HPV- samples (Supplementary Fig. S1D). Taken together, the findings from the two datasets are consistent and highlight the importance of NK cells in HPV- HNSCC.

### Upregulation of IL6/IL6R and CCL2/CCR2 signaling between NK cells and HPV − HNSCC cells in the TME

Next, we performed differential analysis of NK cells between HPV − samples and HPV + samples using the above scRNA-seq data to explore the potential contribution of NK cells to HNSCC immunology. We retrieved the expression data associated to identified NK cells from GSE164690 and GSE139324 using our established labeling. Employing a variant of the t-test, we identified DEGs and generated a list of genes with higher expression (logFC > 1.5, adjusted *p*-value < 0.05) in HPV − samples compared to HPV + samples. In NK cells, 1,043 DEGs from GSE164690 (Supplementary Table S3) and 1,190 DEGs from GSE139324 (Supplementary Table S4), with 450 DEGs overlapping, were identified as highly expressed genes in HPV − samples (*vs.* HPV + samples). We conducted pathway analysis for each set of DEGs using KEGG pathway enrichment tools, where we utilized all the genes listed along with their respective logFC and adjusted *p*-values. For NK cells extracted from the GSE164690 dataset, the five most enriched pathways were ‘viral protein interaction with cytokine and cytokine receptor’, ‘cytokine-cytokine receptor interaction’, ‘toll-like receptor signaling pathway’, ‘rheumatoid arthritis, and malaria’ (Fig. 1J). For NK cells extracted from the GSE139324 dataset, the top five enriched pathways encompassed ‘viral protein interaction with cytokine and cytokine receptor’, ‘cytokine-cytokine receptor interaction’, ‘hematopoietic cell lineage’, ‘chemokine signaling pathway’, and ‘glycine, serine, and threonine metabolism’ (Fig. 1J).

**Fig. 2:**
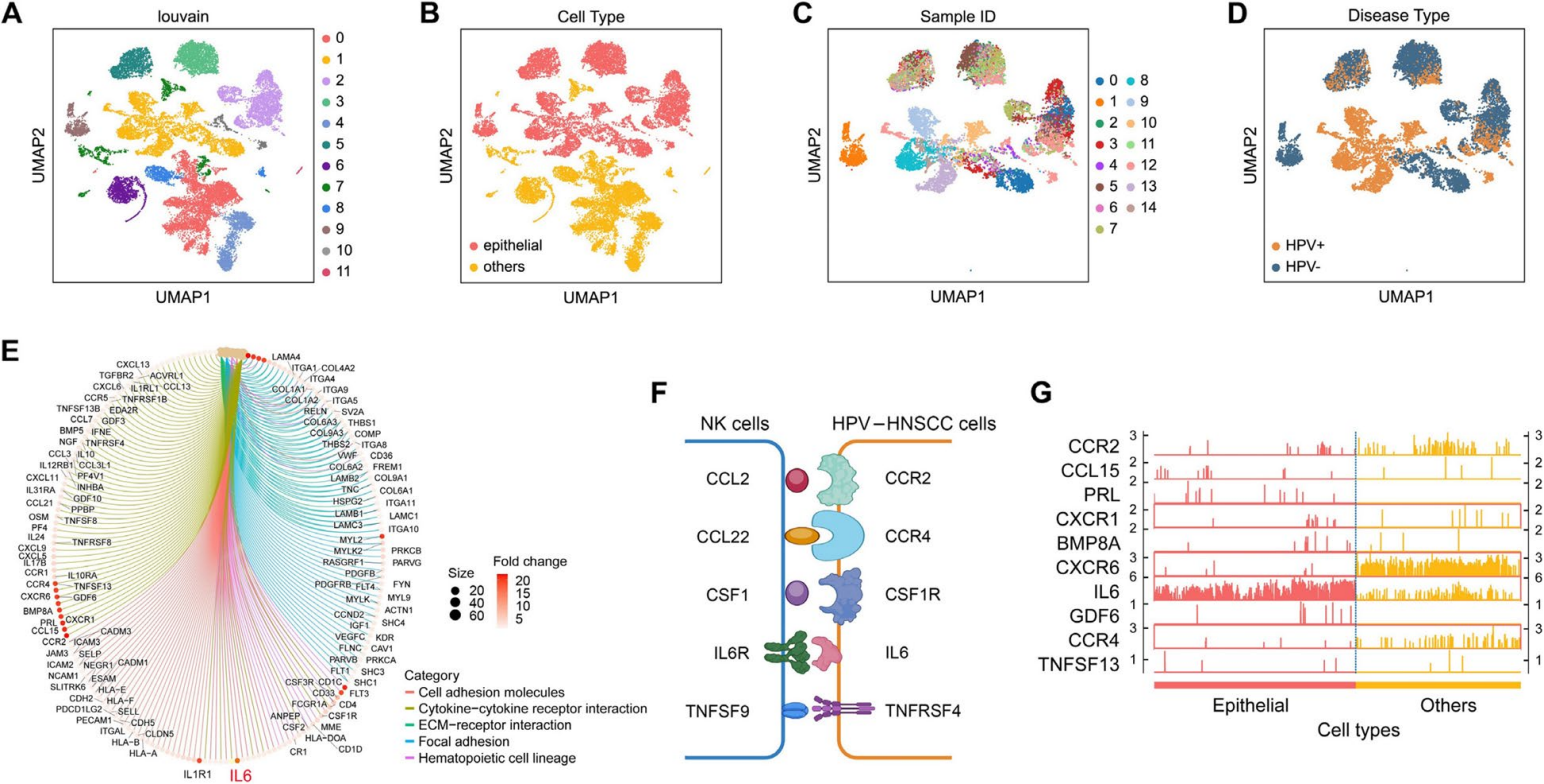
scRNA-seq data analysis identifies DEGs and enriched pathways within NK cells and tumor cells stratified by HPV infection status. Bioinformatic analysis of tumor cells based on the scRNA-seq data from GSE164690. **A** Louvain clustering on all analyzed cells identified 10 cell clusters. **B** Identification of cell types. **C** Visualization of identified tumor cells using UMAP, colored by sample ID. **D** Visualization of identified tumor cells using UMAP, colored by HPV status. **E** Enriched pathways in HPV − HNSCC cells *vs* HPV + HNSCC cells. **F** Five specific gene pairs identified between NK cells and HPV − HNSCC cells. **G** Visualization of the top 10 DEGs in (HPV − *vs* HPV +) in epithelial tumor cells

To relate the biological activities in immune cells with those in non-immune cells, we further analyzed the non-immune cells contained in the GSE164690 dataset. The preceding filtering and pre-processing steps were the same as in the previous analysis of immune cells. After UMAP visualization and checking batch effect, we performed Louvain clustering on all analyzed cells. Louvain clustering identified 12 clusters (Fig. 2A). To extract tumor cells from the cell population, we examined the expression level of a list of marker genes specific for epithelial cells in each cluster. A total of 6 clusters were identified (clusters 1, 2, 3, 5, 9, and 10), in which the marker genes were highly expressed. Cells in these clusters were classified as epithelial cells, or more generally, tumor cells, and were retained for downstream analysis (Fig. 2B). Within the tumor cells (Fig. 2C and D), we again detected the DEGs that were more highly expressed in HPV − samples than in HPV + samples using the same test method and threshold and identified 1,893 DEGs in tumor cells. The top 5 enriched pathways included ‘cell adhesion molecules’, ‘cytokine-cytokine receptor interaction’, ‘ECM-receptor interaction’, ‘focal adhesion’, and ‘hematopoietic cell lineage’ (Fig. 2E).

Of the three sets of DEGs generated by contrasting HPV − tumors with HPV + tumors (Supplementary Table S3, Table S4, and Table S5), two originated from the analysis of NK cells, while the last one stemmed from the analysis of tumor cells. Strikingly, the ‘cytokine-cytokine receptor interaction’ pathway was observed in all three sets among the top 5 enriched pathways, suggesting that this signaling significantly contributes to the dynamic interplay between NK cells and tumor cells within the TME. Within the pathway-specific genes, 10 genes were present in both DEG sets for NK cells, while 65 genes were present in the DEG set for tumor cells. These shared genes exhibited higher expression levels in the HPV − HNSCC cohort and were molecular modulators of the ‘cytokine-cytokine receptor interaction’ pathway. Among them, we found five specific gene pairs: IL6/IL6R, CCR2/CCL2, CCR4/CCL22, CSF1R/CSF1, and TNFRSF4/TNFSF9 (Fig. 2F and Supplementary Fig. S2). Chemokines and cytokines, such as IL6/IL6R and CCR2/CCL2, serve as fundamental regulators within the TME, which has been firmly established as a pivotal driving force in cancer progression [23]. IL6R and CCL2 exhibited elevated expression in NK cells in HPV − tumor tissues compared with HPV + tumor tissues (Fig. 2F). In addition, IL6 and CCR2 were among the top 10 most upregulated DEGs in HPV − tumor cells compared with HPV + tumor cells (Fig. 2G). These findings imply that IL6/IL6R and CCR2/CCL2 signaling pathways are more robustly activated in the microenvironment of HPV − tumors compared to HPV + tumors, which may abrogate the antitumor immune response of NK cells against HPV − tumors.

### IL6 regulates the ‘natural killer cell mediated cytotoxicity’ pathway in TCGA HPV − HNSCC cohort

**Fig. 3:**
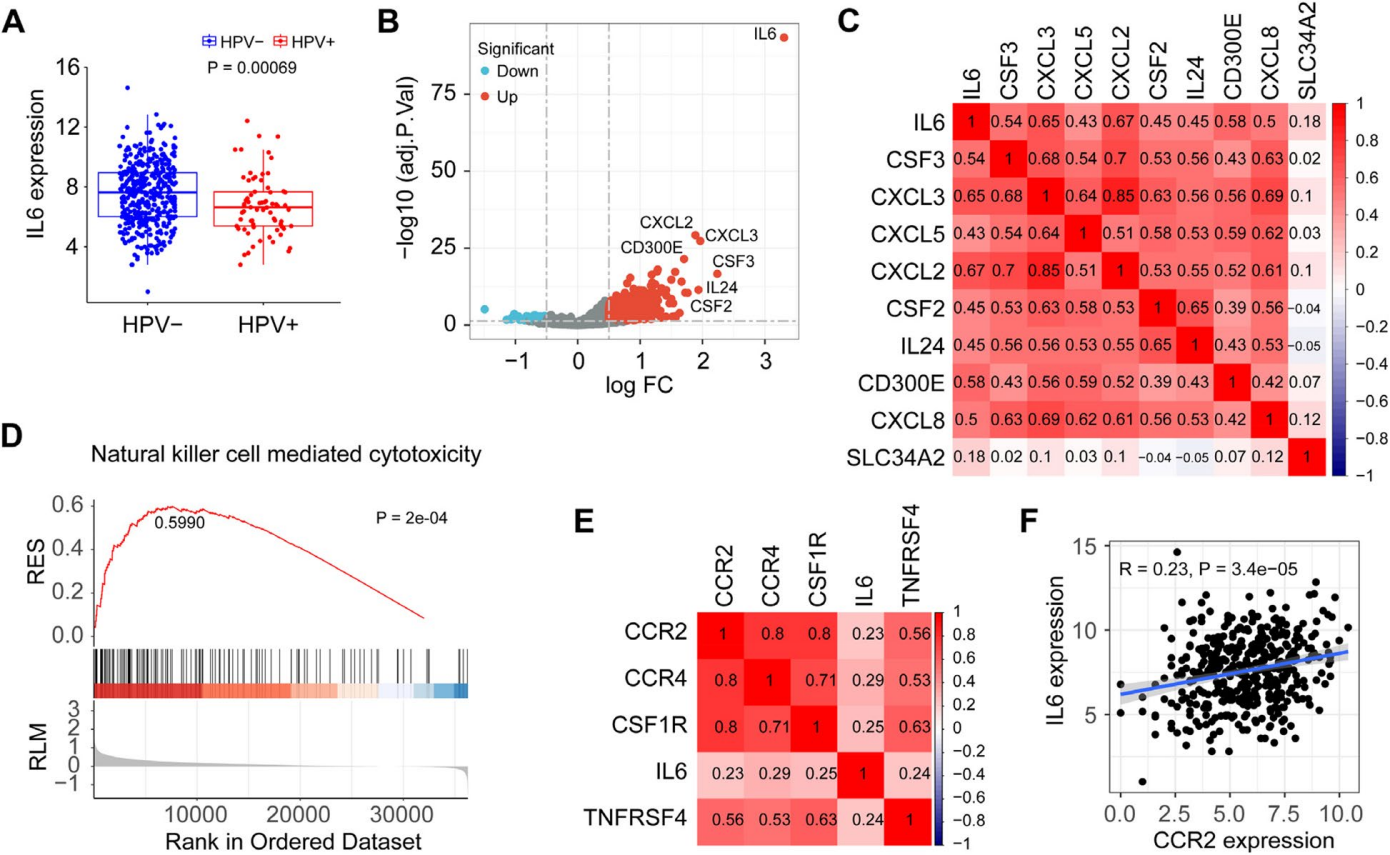
Gene expression and GSEA analysis of IL6 in TCGA HNSCC cohorts. **A** Differential analysis of IL6 expression between HPV − and HPV + HNSCC cohorts using TCGA dataset. **B** DEGs stratified by IL6 high and low expression in HPV − HNSCC cohort. **C** Expression correlation analysis between IL6 and the top nine upregulated DEGs identified in the IL6 high-expression group. **D** Pathway enrichment analysis (IL6 high expression *vs* IL6 low expression) revealing the ‘natural killer cell mediated cytotoxicity’ pathway in TCGA HPV − HNSCC cohort. **E** Correlation between the expression of IL6 and the other 4 DEGs upregulated in HPV − HNSCC cells *vs* HPV + HNSCC cells. **F** Correlation analysis of the gene expression between IL6 and CCR2 in TCGA HPV − HNSCC cohorts

Next, we analyzed IL6 expression using TCGA HNSCC dataset. This analysis showed a significant increase in IL6 in HPV − HNSCC compared with HPV + HNSCC (Fig. 3A). To investigate the role of the IL6 gene in HPV − HNSCC, we performed pathway enrichment analysis of IL6 using single-gene GSEA with TCGA RNA-seq data obtained from the HPV − HNSCC cohort. Nineteen genes, including CSF2, CSF3, CXCL2, CXCL3, CXCL5 and CD300E, were significantly (logFC > 1.5, adjusted *p*-value < 0.05) correlated with the expression of IL6 gene in HPV − tumors (Fig. 3B and Supplementary Table S6). Among them, only co-expression between IL6 and CXCL2/3 had a high correlation (Spearman correlation coefficient > 0.6) in these cases (Fig. 3C). In addition, GSEA revealed a significantly upregulated ‘natural killer cell mediated cytotoxicity’ pathway (enrichment score = 0.5990, adjusted *p*-value = 2e-04) in HPV − HNSCC cases but not in HPV + HNSCC cases (Fig. 3D and Supplementary Table S7 and Table S8), suggesting that IL6 may play an important role in the regulation of NK cell-mediated tumor immunity in the microenvironment of HPV − tumors. Furthermore, co-expression analysis in TCGA HPV − HNSCC cohort revealed that the correlation between IL6 and CCR2 exhibited the lowest value (*R* = 0.23) among the 5 DEGs identified from our scRNA-seq data analysis (Fig. 3E and F).

### Blocking IL6 signaling suppresses the development and progression of HPV − HNSCC in mice

**Fig. 4:**
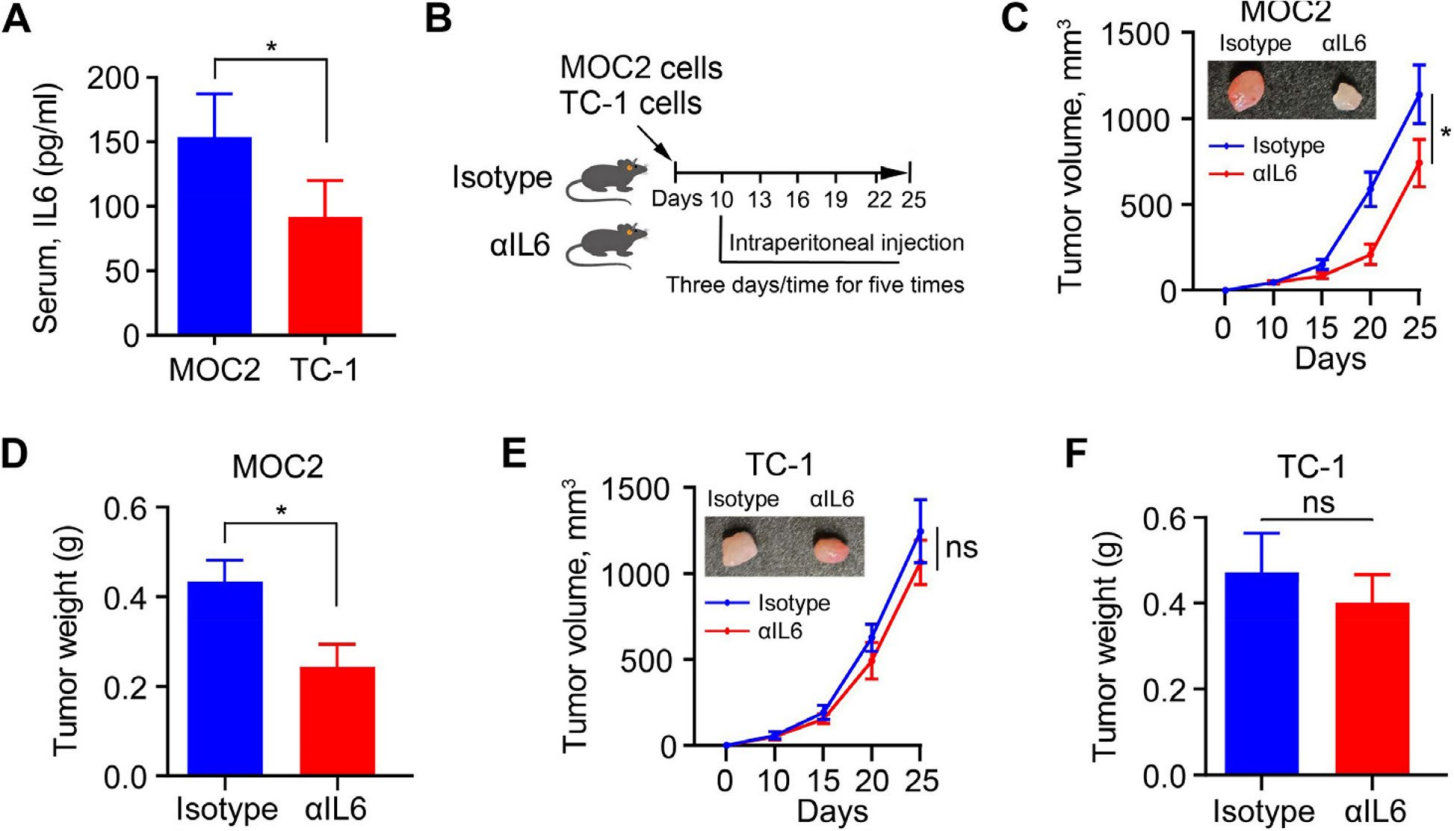
Blocking IL6 signaling induces the remission of HPV − tumors in orthotopic tumor mouse models. **A** IL6 concentration in the peripheral serum of C57BL/6 mice bearing MOC2 or TC-1 tumors. **B** Schematic showing the timeline of experimental procedures. Male C57BL/6 mice at 6–8 weeks of age received intramucosal injection of 1.0 × 10^6^ MOC2 or TC-1 cells. When tumors were established (10 days after cell inoculation), mice were randomized to receive the treatment of IgG2 isotype or αIL6 (100 µg/mouse) (*n* = 5/group). **C**, **D** MOC2 tumor growth curve and weight in each treatment group. **E**, **F** TC-1 tumor growth curve and weight in each treatment group. In (**C**) and (**E**), representative tumors from C57BL/6 mice with indicated treatment are shown in the upper panel. *p < 0.05; ns: not significant

To determine the abundance of IL6 in HPV − and HPV + HNSCC, we established orthotopic tumors by intramucosal injection of HPV − mouse cancer MOC2 cells or HPV + mouse cancer TC-1 cells into C57BL/6 mice and then measured IL6 concentration in the peripheral serum extracted from these tumor models. Ten days after cancer cell inoculation, significantly higher IL6 concentration was observed in mice bearing MOC2 tumors than in mice bearing TC-1 tumors (Fig. 4A), confirming the bioinformatic results (Fig. 2). We then treated these mice with isotype or αIL6 based on the treatment schedule shown in Fig. 4B. At the experimental endpoint, the concentration of IL6 was significantly decreased in the peripheral serum of mice either implanted with MOC2 or TC-1 cells (Supplementary Fig. S3). More importantly, MOC2 tumor burden was remarkably reduced in mice receiving αIL6, as evidenced by reduced tumor volume and weight (Fig. 4C and D). In contrast, there was no noticeable change in tumor burden between TC-1 tumor-bearing mice treated with isotype or αIL6 (Fig. 4E and F), suggesting that blockade of IL6 has the potential to limit development and progression of HPV − HNSCC.

### Blockade of IL6 signaling facilitates tumor infiltration and proliferation of NK cells in HPV − HNSCC in mice

**Fig. 5:**
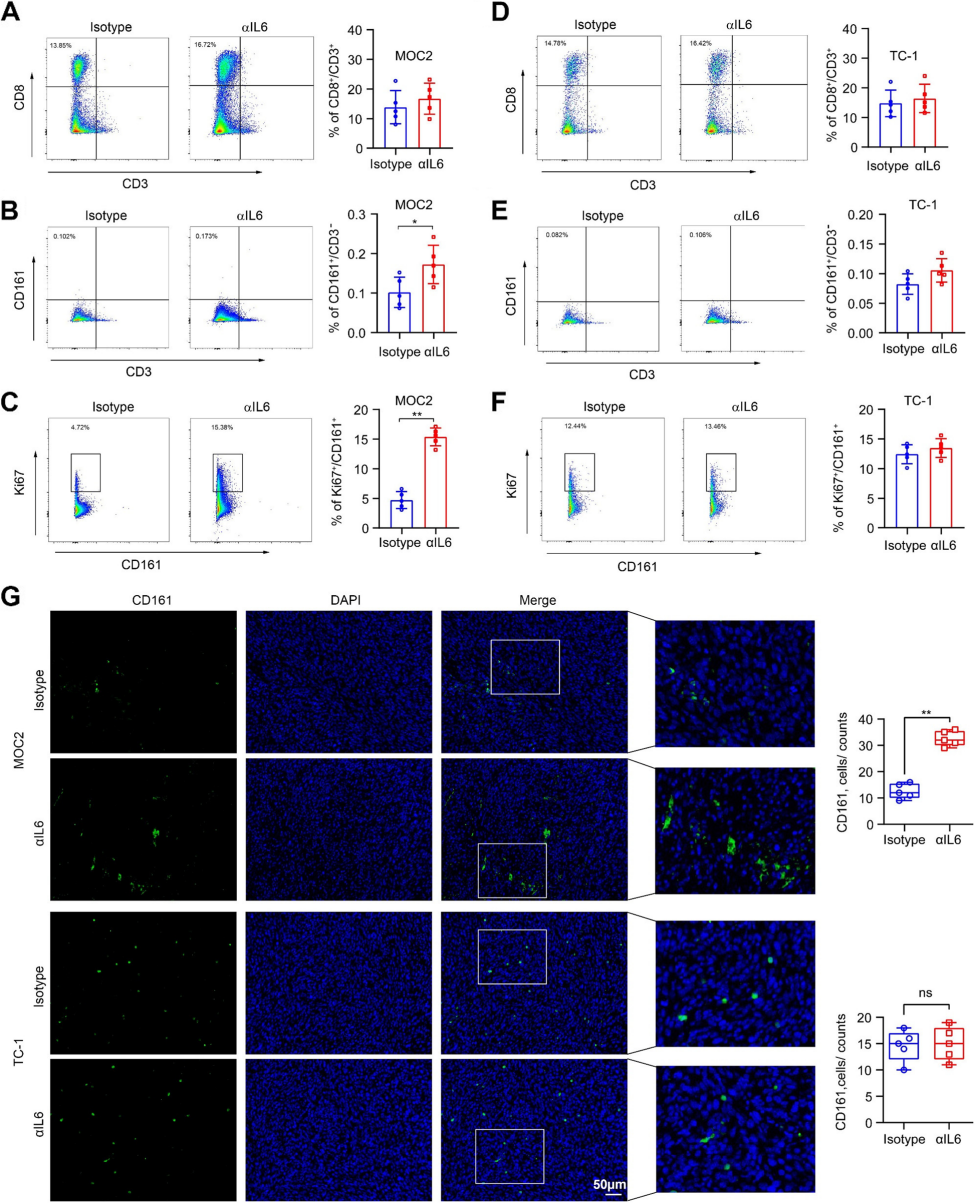
Blocking IL6 signaling enhances the antitumor activity of NK cells in HPV − tumors. **A**, **B** Percent of CD8^+^ T cells and CD161^+^ NK cells in MOC2 tumors treated with isotype or αIL6. **C** Percent of Ki67^+^ NK cells in MOC2 tumors treated with isotype or αIL6. **D**, **E** Percent of CD8^+^ T cells and CD161^+^ NK cells in TC-1 tumors treated with isotype or αIL6. **F** Percent of Ki67^+^ NK cells in TC-1 tumors treated with isotype or αIL6. **G** Immunofluorescence staining of anti-CD161 antibody in MOC2 and TC-1 tumors treated with isotype or αIL6. Representative images and quantitative data are shown in the left and right panels. In (**A**, **B**, **D**, **E**), T cells and NK cells were gated from CD3^+^ and CD3^−^ population, respectively. *p < 0.05; **p < 0.01; ns: not significant

Immune cell tumor infiltration plays a crucial role in tumor immunotherapy. This phenomenon involves the infiltration of various immune cells, such as T cells and NK cells, into the TME. CD161 serves as a marker for NK cells that have maintained their capacity to react to innate cytokines during their differentiation [24, 25]. To investigate the effect of IL6 on tumor infiltration of cytotoxic T cells and NK cells in MOC2 and TC-1 tumors, we determined the number of intratumoral CD8^+^ T cells and CD161^+^ NK cells in the presence or absence of αIL6 treatment. There was no statistically significant difference in the percent of CD8^+^ T cells in MOC2 tumor tissues isolated from mice treated with isotype or αIL6 (Fig. 5A). However, the level of tumor infiltration of CD161^+^ NK cells was significantly increased in αIL6-treated mice compared with isotype-treated mice (Fig. 5B), which was associated with increased proliferative activity in this cell population (Fig. 5C). In TC-1 tumors, there were no discernible differences in the quantities of intratumoral CD8^+^ T cells and CD161^+^ NK cells, nor in the proliferation of NK cells, between mice treated with αIL6 and those treated with the isotype control (Fig. 5D-F). Data from immunofluorescence staining of anti-CD161 antibody in MOC2 and TC-1 tumor tissues confirmed the results from flow cytometry (Fig. 5G), supporting the notion that IL6 signaling contributes more to NK cell tumor infiltration and antitumor efficacy in HPV − HNSCC, and blocking this signaling facilitates tumor infiltration and proliferation of NK cells.

### Combined treatment with αIL6 and RS504393 promotes NK cell antitumor activity and induces HPV − tumor remission more potently than either single agent treatment in mice

**Fig. 6:**
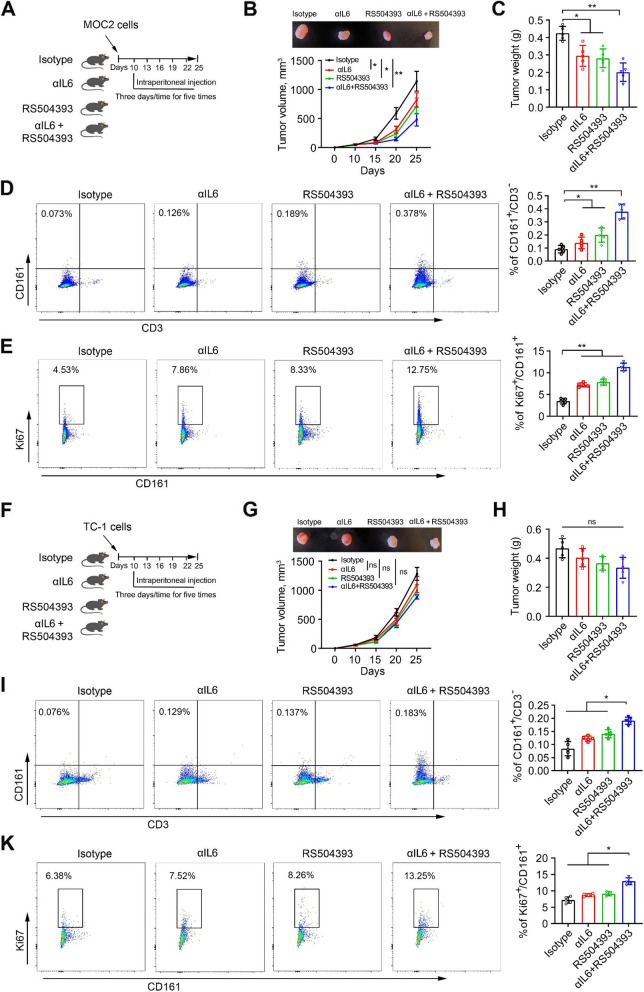
αIL6 and RS504393 promote NK cell antitumor activity and repress MOC2 tumors more effectively when combined. **A**, **F** Schematic showing the timeline of experimental procedures. Male C57BL/6 mice at 6–8 weeks of age received intramucosal injection of 1.0 × 10^6^ MOC2 or TC-1 cells. When tumors were established (10 days after cell inoculation), mice were randomized to receive the treatment of IgG2 isotype, αIL6 (100 µg/mouse), RS504393 (6 mg/kg) or the combination of αIL6 and RS504393 (*n* = 5/group). **B**, **C** MOC2 tumor growth curve and weight in each treatment group. Representative tumors from C57BL/6 mice with indicated treatment are shown in the upper panel of (**B**). **D**, **I** Percent of CD161^+^ NK cells in MOC2 (**D**) or TC-1 (**I**) tumors treated with αIL6 and RS504393 alone or in combination. Representative images and quantitative data are shown in the left and right panels. **E**, **K** Percent of Ki67^+^ NK cells in MOC2 (**E**) or TC-1 (**K**) tumors treated with αIL6 and RS504393 alone or in combination. Representative images and quantitative data are shown in the left and right panels. **G**, **H** TC-1 tumor growth curve and weight in each treatment group. Representative tumors from C57BL/6 mice with indicated treatment are shown in the upper panel of (**G**). *p < 0.05; **p < 0.01; ns: not significant

Based on our scRNA-seq data analysis, the antitumor activity of NK cells may also be regulated by other pathways, such as CCL2/CCR2 signaling axis (Fig. 2F). The least significant expression correlation among the 5 DEGs (IL6, CCR2, CCR4, CSF1R, and TNFRSF4) identified in HPV − HNSCC cells was observed between IL6 and CCR2 (Fig. 3E). This finding suggested that the CCR2 and IL6 signaling axis might not be dependent on each other, and co-inhibition of them could potentially produce a synergistic treatment effect. Based on this, we assessed the therapeutic efficacy of double blockade of IL6 and CCR2 signaling in orthotopic tumor models. RS504393 is an extremely selective CCR2 chemokine receptor antagonist [26, 27]. To evaluate the combined effectiveness of αIL6 and RS504393 in vivo, MOC2 tumor-bearing mice were treated with αIL6 and RS504393 alone or in combination. Following five treatment cycles, there was a noticeable decrease in tumor burden for each individual treatment agent. This was supported by the observation of smaller tumor size and reduced tumor weight when compared to mice treated with the isotype control (Fig. 6B and C). Remarkably, the concurrent administration of αIL6 and RS504393 produced an anticancer outcome that surpassed the efficacy of either treatment alone (Fig. 6B and C), leading to an augmentation of tumor-infiltrating NK cells, along with enhanced NK cell proliferation (Fig. 6D and E). The combination of αIL6 and RS504393 also resulted in an increase in tumor infiltration and proliferation of NK cells in HPV + TC-1 tumors compared with the other three groups; however, this combination did not show any antitumor activity in these tumors (Fig. 6F-K). We also determined the effect of the αIL6/RS504393 combination on tumor infiltration of cytotoxic T cells. Clearly, this combination did not lead to a significant change in the number of tumor-infiltrating CD8^+^ cells in either MOC2 tumors or TC-1 tumors (Supplementary Fig. S4), suggesting that αIL6 and RS504393 alone or their combination primarily act on NK cells in the TME.

**Fig. 7:**
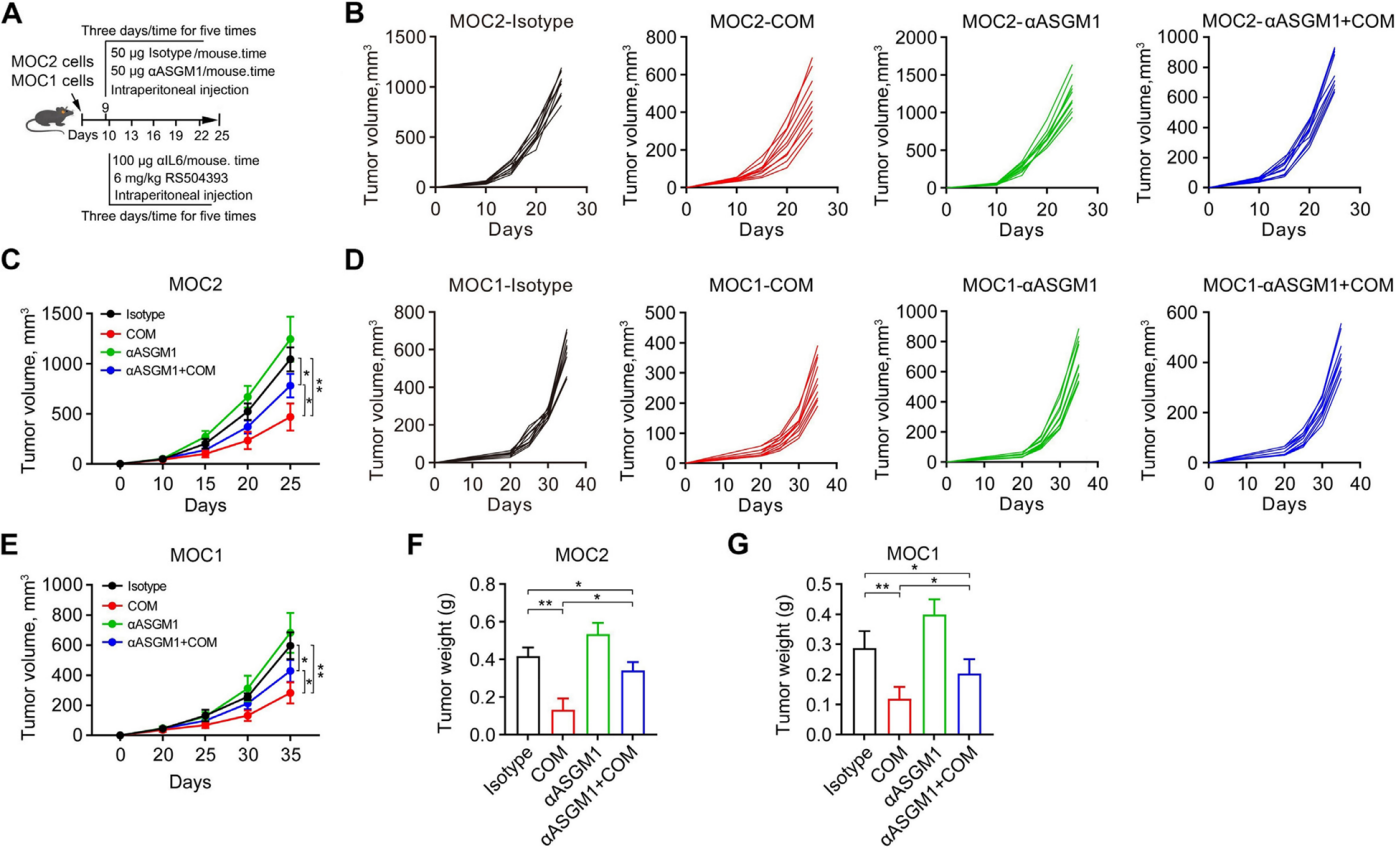
Depletion of NK cells attenuates the antitumor activity of the αIL6 and RS504393 combination in orthotopic mouse models. **A** Schematic showing the timeline of experimental procedures. Male C57BL/6 mice at 6–8 weeks of age received intramucosal injection of either 1.0 × 10^6^ MOC2 or MOC1 cells. Tumor-bearing mice were randomized to receive the combination treatment of the αIL6 (100 µg/mouse) and RS504393 (6 mg/kg), in the presence of αASGM1 or rabbit polyclonal IgG isotype (*n* = 10/group). **B**, **C** Individual and average MOC2 tumor growth curves. **D**, **E** Individual and average MOC1 tumor growth curves. **F**, **G** MOC2 and MOC1 tumor weight in each treatment group. *p < 0.05; **p < 0.01; ns: not significant

To further investigate the contribution of NK cells to the therapeutic efficacy of IL6/CCR2 double blockade, we depleted NK cells in C57BL/6 mice by repeated injections of αASGM1 (Fig. 7A). Depletion of NK cells did not affect MOC2 tumor growth (Fig. 7B and C), but strikingly, αASGM1 significantly reduced the growth difference between primary tumors treated with or without the combination of αIL6 and RS504393 (Fig. 7B and 7C). Similar trends were noted in the buccal mucosal MOC1 tumor models (Fig. 7D and E), providing further confirmation of the role of NK cells in the antitumor immune response induced by the combination of αIL6 and RS504393. The tumor weight results from the indicated treatment groups supported the findings observed in the tumor growth curve analysis (Fig. 7F and G). Additionally, our data showed that αASGM1 did not fully negate the antitumor effectiveness of the combined treatment with αIL6 and RS504393, suggesting that the synergistic effects observed with the combination of αIL6 and RS504393 may also occur through a mechanism independent of NK cells.

**Fig. 8:**
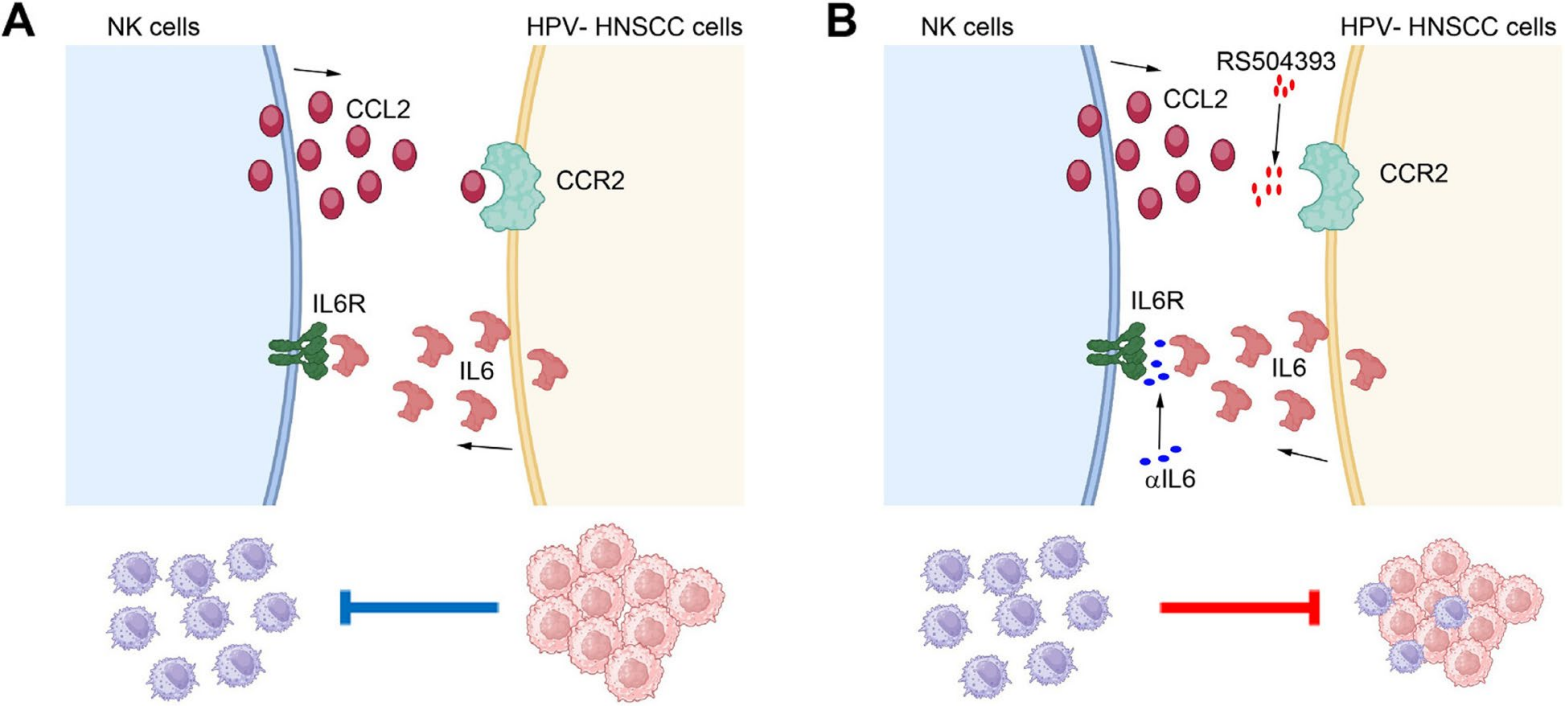
A proposed model for this study. **A** In the microenvironment of HPV − HNSCC, IL6/IL6R and CCL2/CCR2 signaling exert a significant influence on the ability of HPV − tumor cells to evade immune attacks by NK cells. **B** Simultaneous blockade of IL6/IL6R and CCL2/CCR2 with αIL6 and RS504393 is effective in enhancing the antitumor activity of NK cells in HPV − HNSCC

Taken together, these findings provide compelling evidence that within the immunosuppressive TME, the impact of the IL6/IL6R and CCL2/CCR2 signaling on the ability of HPV − tumors to evade immune attack by NK cells is more pronounced than their impact on HPV + tumors, and dual blockade of these two pathways is more effective to potentiate the antitumor activity of NK cells in HPV − HNSCC cells (Fig. 8).

## Discussion

Patients with HPV + HNSCC exhibit markedly improved survival rates after undergoing standard chemoradiation therapy (CRT) or immunotherapy in contrast to individuals with HPV − HNSCC [28, 29]. The differential response, at least in part, is attributed to the TME. HPV infection induces alterations in immune cell infiltration in HNSCC, creating a diverse and heterogeneous immune landscape [1, 30]. In the case of HPV + HNSCC, viral proteins act as foreign antigens, alongside neoantigens resulting from viral integration and mutagenesis induced by the viral restriction factor APOBEC3 [29]. While HPV − HNSCC lacks these foreign antigens, which emerge from either extensive random mutations or the overexpression of cellular genes [31]. The immunological response to viral antigens may contribute to the more favorable clinical course, as the TME in HPV + tumors is more enriched than that in HPV − tumors. PD-1-targeted ICIs that unleash T cells against recurrent HNSCC have produced remarkable results in some patients, but these agents fail in the majority of patients with HPV − HNSCC. There is a growing enthusiasm for exploring additional immune effector cells, including NK cells, as potential targets for immunotherapy.

A retrospective analysis of a randomly selected cohort of 140 oropharyngeal squamous cell carcinoma (OSCC) cases revealed a higher proportion of cytotoxic NK cells in HPV + OSCC compared to HPV − OSCC [32]. In contrast, our findings showed that the proportion of NK cells in cluster 4 was higher in HPV − HNSCC compared with HPV + HNSCC (0.073 *vs.* 0.024 in GEO164690 and 0.069 *vs.* 0.014 in GEO139324). There are several potential reasons for the discrepancies observed between the two studies. The discrepancies in NK cell numbers in HPV + and HPV- subtypes may be attributed, primarily, to variations in patient cohorts. HNSCC is a heterogeneous group of tumors involving distinct anatomical sites and subsites with varying etiological factors including smoking and alcohol use. Second, the samples collected from patients may vary in terms of sex, age, and tumor stage, all of which can influence the presence of NK cells. Third, the differences in results may indeed be due to the different methodologies used in the two studies. Wagner and colleagues obtained their results from a combination of immunohistochemistry and PCR followed by bead-based hybridization using Luminex technology [32]. While our results were derived from scRNA-seq data. The scRNA-seq approach enables a detailed examination of immune responses at the single-cell level, providing a comprehensive view of interactions among different immune cell types and other cell populations within the tumor microenvironment (TME) during immune responses. Fourth, Wagner et al. drew conclusions based on CD56 + lymphocytes [32], While we identified the NK cell cluster based on a set of NK cell marker genes. CD56 is a classic phenotypic marker of natural killer cells; however, it is expressed by a variety of immune cells, including alpha–beta T cells, gamma-delta T cells, dendritic cells, and monocytes [33, 34]. Finally, Wagner’s group focused only on the cytotoxic NK cell subpopulation [32], whereas all types of NK cells were included in our scRNA-seq analysis. CD56^+^ NK cells can be divided into CD56^dim^CD16^+^ cells, which are more cytotoxic, and CD56^bright^CD16^−^ cells, which produce high levels of cytokines such as IFNγ and TNFα [35, 36]. Further confirmation from additional cohorts and alternative approaches is necessary to comprehensively investigate the impact of HPV infection status on the number of intratumoral NK cells. Nevertheless, little is known about the interplay between NK cells and tumor cells that differ by HPV infection status. In the present study, by integrating scRNA-seq data with TCGA HNSCC dataset, we identified five enriched pathways, including IL6/IL6R and CCL2/CCR2 signaling, within NK cells and tumor cells in the HPV − HNSCC cohort compared to the HPV + counterpart. These pathways are promising as potential signaling nodes to precisely target the regulatory network between NK cells and HPV − tumor cells. Aligned with this notion, we found that blocking IL6 signaling with αIL6 was only effective in mice bearing HPV − tumors.

The chemokine CCL2 and its primary receptor CCR2 have garnered significant attention due to their involvement in cancer development. Upregulation of CCL2/CCR2 and various immune conditions in prostate cancer are associated with cancer progression, metastasis, and relapse [37]. The CCL2/CCR2 signaling axis also recruits various immune cells to form an immunosuppressive TME in both the early and late stages of metastasis, which allows tumor cells to evade immune surveillance [38]. Another study reported that CCL2 induced by hypoxic tumor cells can inhibit the maturation of NK cells in the pre‐metastatic niche and reduce their ability to eliminate incoming circulating tumor cells, thereby accelerating the homing of tumor cells to CCR2 [39]. Results from our bioinformatic study revealed CCL2/CCR2 as one of the five pathways most upregulated within NK cells and HPV − tumor cells (*vs.* HPV + tumor cells). Given the lack of significant interdependence between the IL6 and CCR2 pathways, we chose to combine αIL6 with RS504393 for the treatment of HPV − HNSCC. RS504393 is a selective CCR2 antagonist, which has shown strong antitumor activity in the mouse model of bladder cancer [26]. As expected, dual IL6 and CCR2 blockade reduced the tumor burden in an orthotopic mouse model of HPV − tumors more efficiently than either agent alone. Our data also indicate increased antitumor activity of NK cells, but not CD8^+^ T cells, upon neutralizing IL6 alongside CCR2 blockade, which is distinct from PD-1/PD-L1 blockade. While targeting both IL6 and CCR2 appears to be a promising therapeutic approach for HPV − HNSCC, it is critical to recognize that factors beyond NK cell stimulation may contribute to the divergent response to IL6/CCR2 double blockade between HPV − and HPV + tumors. Further molecular investigations and in-depth mechanistic studies are warranted to unravel this complexity.

Recent studies have highlighted that NKG2A as a pivotal checkpoint for the activation of tumor infiltrating cytotoxic CD8^+^ T cells and NK cells in cancerous conditions [8]. Blocking antibodies, such as monalizumab (IPH2201, a first-in-class ICI that targets the NKG2A receptors), to NKG2A unleashed the reactivity of these effector cells resulting in tumor control in multiple mouse models and an early clinical trial [40–42]. Nevertheless, the clinical trial results from the phase 3 INTERLINK-1 trials (NCT04590963) indicated a lack of therapeutic benefit when combining monalizumab with cetuximab in patients with HPV − HNSCC. This setback in monalizumab-based therapy in HNSCC underscores the need for an alternative treatment to enhance the antitumor effect of NK cells. The limited presence of NK cells in HNSCC contributes to the formation of an immunosuppressive TME. Our results indicate that selective blockade of both IL6 and CCR2 pathways can also enhance NK cell infiltration and proliferation in HPV + tumors. However, the phenotypes observed in HPV + tumors differ from those observed in HPV − tumors, where blockade of either pathway alone is sufficient to enhance NK cell recruitment and proliferation. At present, the mechanisms underlying these phenotypes remain unclear. Our study also provided evidence that the IL6/IL6R and CCL2/CCR2 signaling pathways within the TME exert a greater influence on the ability of HPV − tumor cells to evade immune attack by NK cells compared to HPV + tumors. Therefore, simultaneous blockade of IL6 and CCR2 appears to be a promising therapeutic approach for the treatment of HPV − tumors by augmenting the tumor-infiltrating NK cell population. Nevertheless, we recognize the need to further explore the underlying molecular mechanisms for a more in-depth investigation. In addition, the preclinical data on the dual blockade of IL6 and CCR2 in a broader range of HPV − HNSCC cell lines are essential before moving into clinical trials.

In conclusion, novel immune-based therapies are highly dependent on a thorough understanding of the interactions between tumor cells and immune cells. This study clearly demonstrates distinct aspects of molecular signaling within NK cells and tumor cells between HPV + and HPV − HNSCC subtypes, thereby laying the foundation for the incorporation of various immune-oriented therapies in the treatment of HPV-unrelated HNSCC.

## Conclusions

Coupled with rigorous scRNA-seq analysis and experimental validation in highly clinically relevant animal models, our work has demonstrated that simultaneous blockade of IL6 and CCR2 is a potential approach to enhance the antitumor activity of NK cells in HPV − HNSCC, providing a scientific rationale for the development of more effective immunotherapy-based treatments for this disease subset.

### Supplementary Information

**Supplementary Material 1.**

**Supplementary Material 2.**

**Supplementary Material 3.**

**Supplementary Material 4.**

**Supplementary Material 5.**

**Supplementary Material 6.**

**Supplementary Material 7.**

## Data Availability

Processed feature barcode matrices for all scRNA-seq data are available on the GEO database with accession number GSE164690 and GSE139324. Gene expression and clinical data are available on the TCGA HNSCC database (https://portal.gdc.cancer.gov/repository). This paper does not report original code. Any additional information required to reanalyze the scRNA-seq data reported in this paper is available from the lead contact upon request. Other data generated or analyzed during this study are included in this published article [and its supplementary information files].

## Abbreviations

αASGM1: Anti-Asialo GM1 antibody
CCL2: C-C motif chemokine ligand 2
CCR2: C-C motif chemokine receptor 2
CRT: Chemoradiation therapy
FC: Fold change
GO: Gene Ontology
GEO: Gene Expression Omnibus
GSEA: Gene Set Enrichment Analysis
HLA-A: Major histocompatibility complex, class I, A
HLA-B: Major histocompatibility complex, class I, B
HLA-C: Major histocompatibility complex, class I, C
HLA-F: Major histocompatibility complex, class I, F
HLA-G: Major histocompatibility complex, class I, G
HNSCC: Head and neck squamous cell carcinoma
HPV: Human papillomavirus
KEGG: Kyoto Encyclopedia of Genes and Genomes
MOC2: Mouse oral squamous cell carcinoma
NK cells: Natural killer cells
NKG2A: CD94/NK group 2 member A
IL6: Interleukin 6
IL6R: Interleukin 6 receptor
OSCC: Oropharyngeal squamous cell carcinoma
PCA: Principal Component Analysis
TME: Tumor microenvironment
TCGA: The Cancer Genome Atlas
UMAP: Uniform Manifold Approximation and Projection
