# Supplementary material for: Combined IL6 and CCR2 blockade potentiates antitumor activity of NK cells in HPV-negative head and neck cancer
Source: J Exp Clin Cancer Res. 2024 Mar 12;43:76. doi: 10.1186/s13046-024-03002-1 (PMC10929116; doi:10.1186/s13046-024-03002-1)
Supplement: Supplementary file 1 — Supplementary Material 1. [file 13046_2024_3002_MOESM1_ESM.docx]

Supplementary Materials for

**Combined IL6 and CCR2 Blockade Potentiates Antitumor Activity of NK Cells in HPV-Negative Head and Neck Cancer**

Yang F *et al*

Corresponding author: Yong Teng, yong.teng@emory.edu

This PDF file includes:

**Supplementary Tables S1 to S8 and Supplementary Figures S1 to S4**

**Table captions:**

**Table S1. HNSCC Patient cohort information of GSE164690**

**Table S2. HNSCC Patient cohort information of GSE139324**

**Table S3. DEGs in NK cells based on GSE164690 scRNA-seq data (see attached Excel)**

**Table S4. DEGs in NK cells based on GSE139324 scRNA-seq data (see attached Excel)**

**Table S5. DEGs in tumor cells (HPV− *vs.* HPV+) based on GSE164690 scRNA-seq data (see attached Excel)**

**Table S6. Nineteen genes significantly co-expressed with IL6 in HPV- HNSCC cohort (see attached Excel)**

**Table S7. List of significantly altered pathways in IL6 high expressing HPV−** **HNSCC *vs.* IL6 low expressing HPV−** **HNSCC (see attached Excel)**

**Table S8. List of significantly altered pathways in IL6 high expressing HPV+** **HNSCC *vs.* IL6 low expressing HPV+** **HNSCC (see attached Excel)**

**Table S1. HNSCC patient cohort information of GSE164690**

| **Patient ID** | **Gender** | **Age group** | **Smoking** | **Alcohol** | **Disease site** | **HPV status** |
| --- | --- | --- | --- | --- | --- | --- |
| **1** | Male | 70-79 | Yes | No | Oral cavity | Negative |
| **2** | Female | 60-69 | No | No | Oral cavity | Negative |
| **3** | Male | 80-89 | No | No | Oral cavity | Negative |
| **4** | Male | 50-59 | Yes | Yes | Oral cavity | Negative |
| **5** | Female | 50-59 | Yes | Yes | Oral cavity | Negative |
| **6** | Male | 30-39 | Yes | Yes | Oral cavity | Negative |
| **7** | Male | 60-69 | Yes | Yes | Larynx | Negative |
| **8** | Female | 70-79 | Yes | Yes | Oral cavity | Negative |
| **9** | Female | 70-79 | Yes | Yes | Oral cavity | Negative |
| **10** | Male | 50-59 | No | Yes | Oral cavity | Negative |
| **11** | Male | 80-89 | No | No | Oral cavity | Negative |
| **12** | Male | 50-59 | Yes | Yes | Oropharynx | Positive |
| **13** | Male | 70-79 | No | No | Oropharynx | Positive |
| **14** | Male | 50-59 | Yes | Yes | Oropharynx | Positive |
| **15** | Female | 60-69 | Yes | NA | Oral cavity | Negative |
| **16** | Male | 40-49 | Yes | Yes | Oropharynx | Positive |
| **17** | Male | 50-59 | Yes | Yes | Oropharynx | Positive |
| **18** | Male | 50-59 | Yes | Yes | Oropharynx | Positive |

**Table S2. HNSCC patient cohort information of GSE139324**

| **Patient ID** | **Gender** | **Age** | **Smoking** | **Alcohol** | **Disease site** | **HPV status** |
| --- | --- | --- | --- | --- | --- | --- |
| **1** | Male | 45 | Yes | Yes | Oral cavity | Negative |
| **2** | Male | 50 | Yes | Yes | Floor of mouth | Negative |
| **3** | Female | 66 | Former | Unknown | Floor of mouth | Negative |
| **4** | Male | 48 | Yes | Yes | Buccal mucosa | Negative |
| **5** | Male | 45 | Yes | Occasional | Tongue | Negative |
| **6** | Male | 43 | Yes | No | Larynx | Negative |
| **7** | Male | 74 | Yes | No | Lower gum | Negative |
| **8** | Female | 60 | No | No | Tongue | Negative |
| **9** | Male | 80 | No | No | Oral cavity | Negative |
| **10** | Male | 56 | Yes | Yes | Tongue | Negative |
| **11** | Female | 57 | Yes | No | Tongue | Negative |
| **12** | Male | 35 | No | No | Tongue | Negative |
| **13** | Male | 62 | Yes | Yes | Supraglottis | Negative |
| **14** | Female | 77 | Yes | No | Mandable | Negative |
| **15** | Female | 75 | Yes | Yes | Tongue | Negative |
| **16** | Male | 55 | No | Yes | Tongue | Negative |
| **17** | Male | 80 | No | No | Buccal mucosa | Negative |
| **18** | Female | 69 | Yes | Yes | Floor of mouth | Positive |
| **19** | Male | 68 | No | Occassional | Base of tongue | Positive |
| **20** | Male | 62 | Former | Occassional | Base of tongue | Positive |
| **21** | Male | 49 | Former | Unknown | Base of tongue | Positive |
| **22** | Male | 66 | No | Occasional | Tonsil | Positive |
| **23** | Male | 61 | Yes | Yes | Tongue | Positive |
| **24** | Male | 52 | Former | No | Base of tongue | Positive |
| **25** | Male | 75 | No | No | Tonsil | Positive |
| **26** | Male | 59 | Yes | No | Tonsil | Positive |


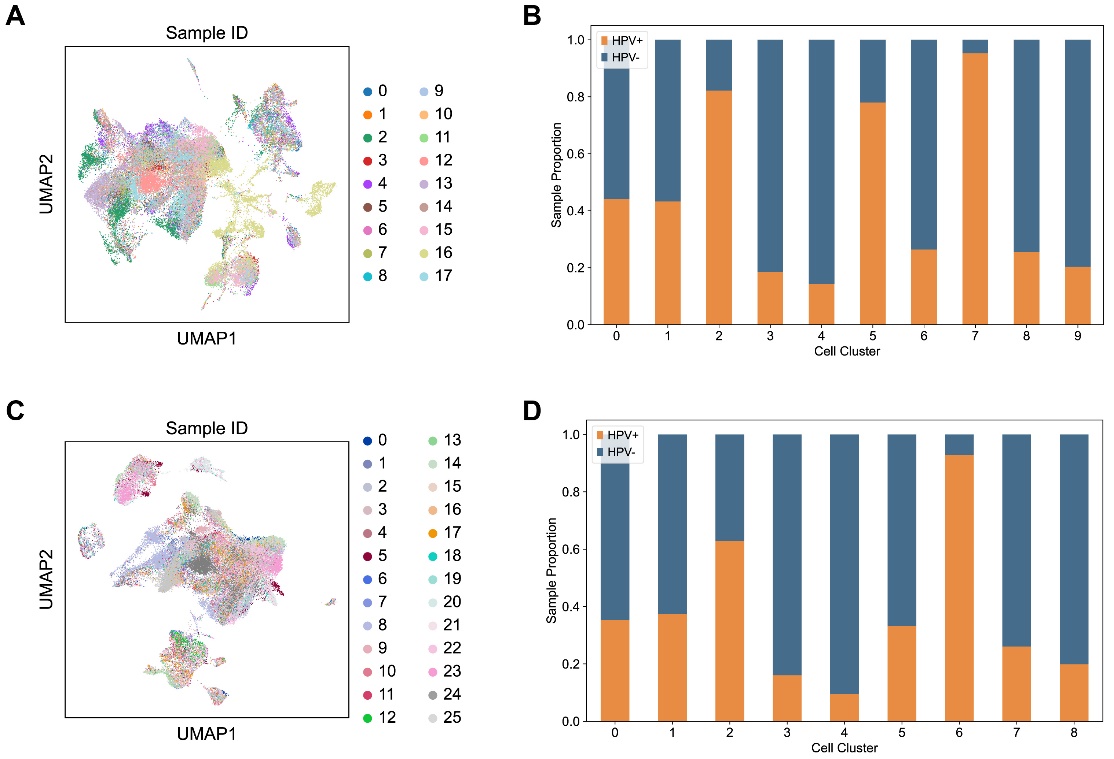


**Figure S1. Bioinformatic analysis of immune cells based on the scRNA-seq data from GSE164690 and GSE139324.** (A) Visualization of all analyzed cells using UMAP, colored by sample ID (GSE164690). (B) Proportion of HPV− samples and HPV+ samples in each cell cluster. (B) Proportion of HPV− samples and HPV+ samples in each cell cluster (GSE164690). (C) Visualization of all analyzed cells using UMAP, colored by sample ID (GSE139324). (D) Proportion of HPV− samples and HPV+ samples in each cell cluster (GSE139324).


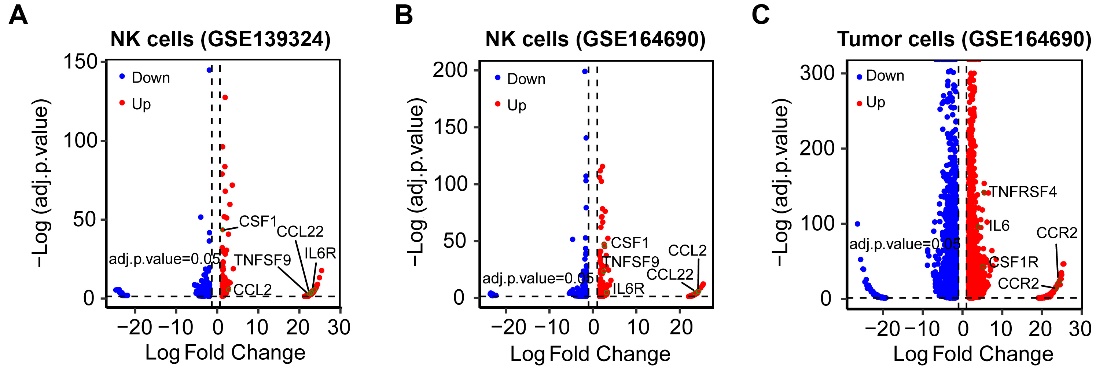


**Figure S2. Volcano plots comparing DEGs in NK cells and tumor cells between the HPV− and HPV+ HNSCC cohorts.** (A) Volcano plots of DEGs in NK cells in the HPV− HNSCC cohort *vs.* the HPV+ HNSCC cohort based on the GSE139324 and GSE164690 scRNA-seq data. (B) A volcano plot of DEGs in tumor cells in the HPV− HNSCC cohort *vs.* the HPV+ HNSCC cohort based on the GSE164690 scRNA-seq data.

**
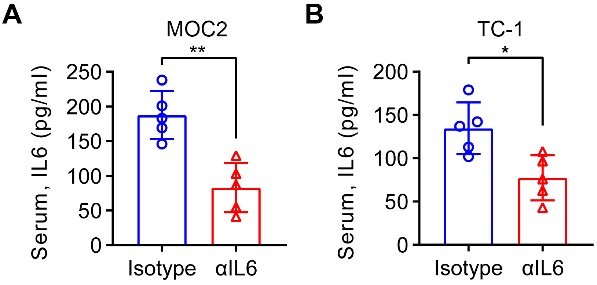
**

**Figure S3. Changes in serum IL6 concentration in tumor-bearing C57BL/6 mice treated with αIL6 or IgG isotype.** The quantitative data are shown from mice bearing MOC2 (A) or TC-1 (B) tumors. ***p*<0.01.


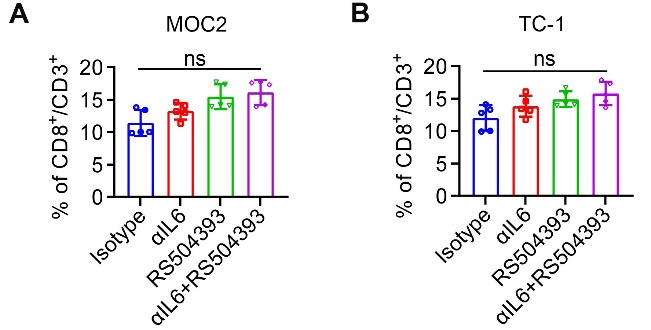


**Figure S4. Percent of CD8^+^ T cells in mouse tumors treated with αIL6 and RS504393, alone or in combination.** T cells were gated from live CD3^+^ population. Quantitative data are shown from mice bearing MOC2 (A) or TC-1 (B) tumors. ns: not significant.
